# Supplementary material for: Sequences of Alterations in Inflammation and Autophagy Processes in Rd1 Mice
Source: Biomolecules. 2023 Aug 22;13(9):1277. doi: 10.3390/biom13091277 (PMC10527025; doi:10.3390/biom13091277)
Supplement: Supplementary file 1 [file biomolecules-13-01277-s001.zip › Supplementary Figure S1.pdf]

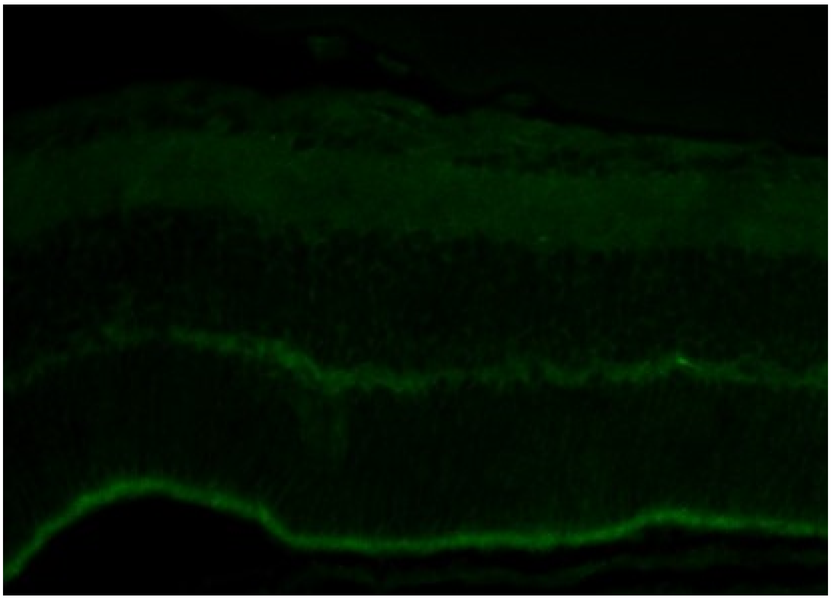

Supplementary Figure S1. Microglia expression in control and rdl retinas. Immunohistochemical images of retinal sections that underwent immunostaining only using the secondary antibody (negative control) and showing the absence of Ibal detection.
