# Supplementary figures and images for: Sequences of Alterations in Inflammation and Autophagy Processes in Rd1 Mice
Source: Biomolecules. 2023 Aug 22;13(9):1277. doi: 10.3390/biom13091277 (PMC10527025; doi:10.3390/biom13091277)

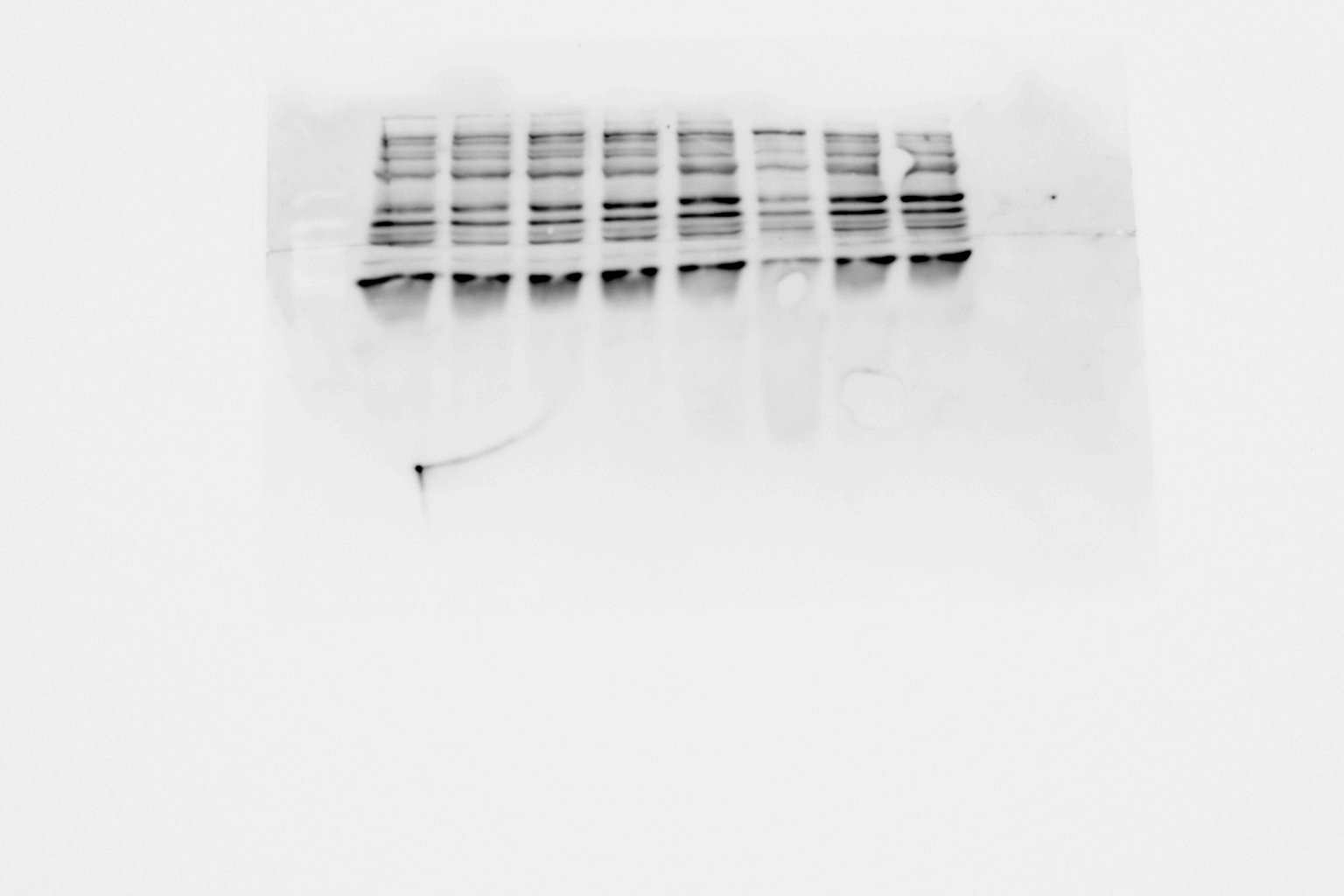

Supplement: Supplementary file 1 [file biomolecules-13-01277-s001.zip › Supplementary File S1/p35 y p42 Atg5/20180113_1436_4.jpg]

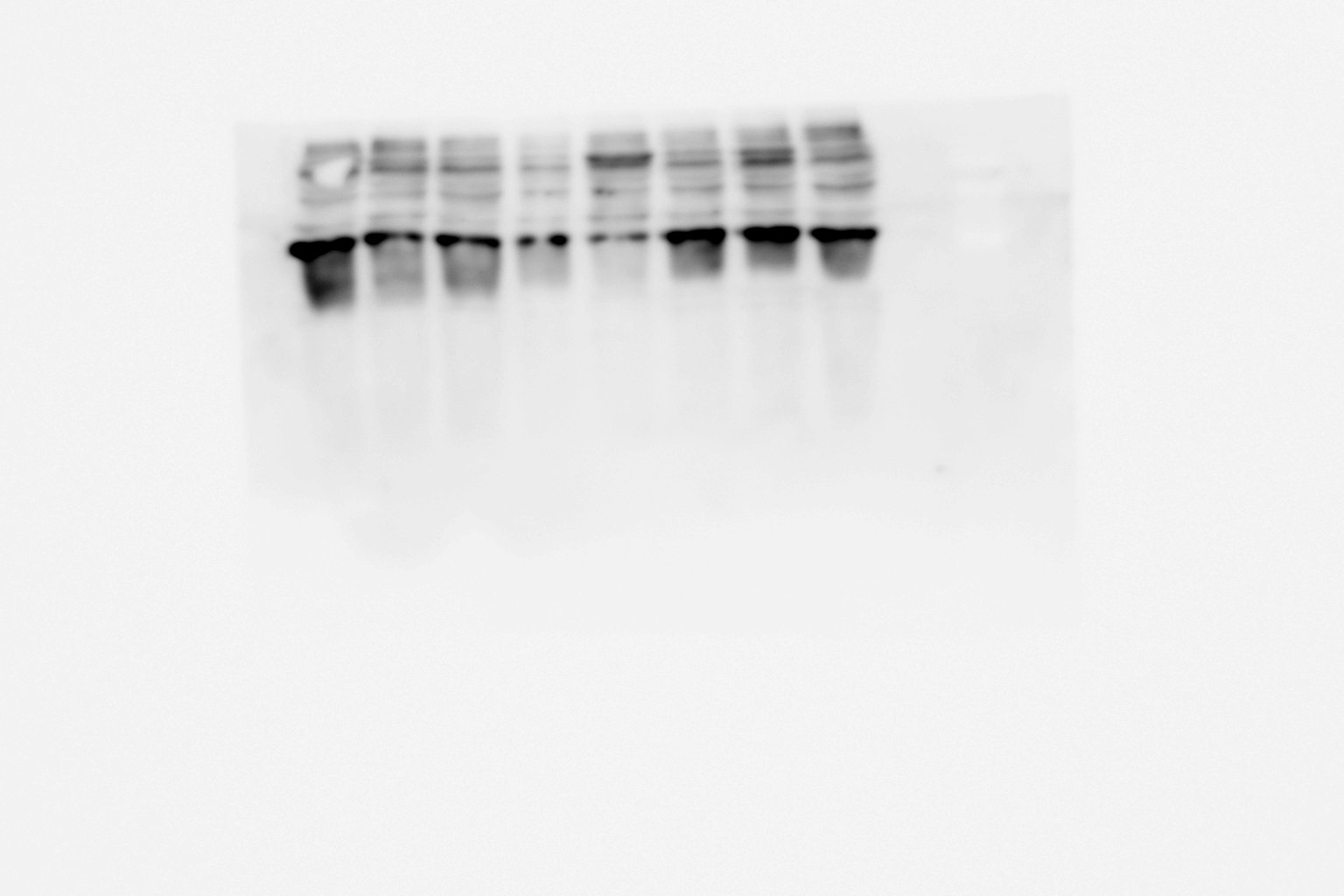

Supplement: Supplementary file 1 [file biomolecules-13-01277-s001.zip › Supplementary File S1/p35 y p42 Atg5/V_20180112_1837_5.jpg]

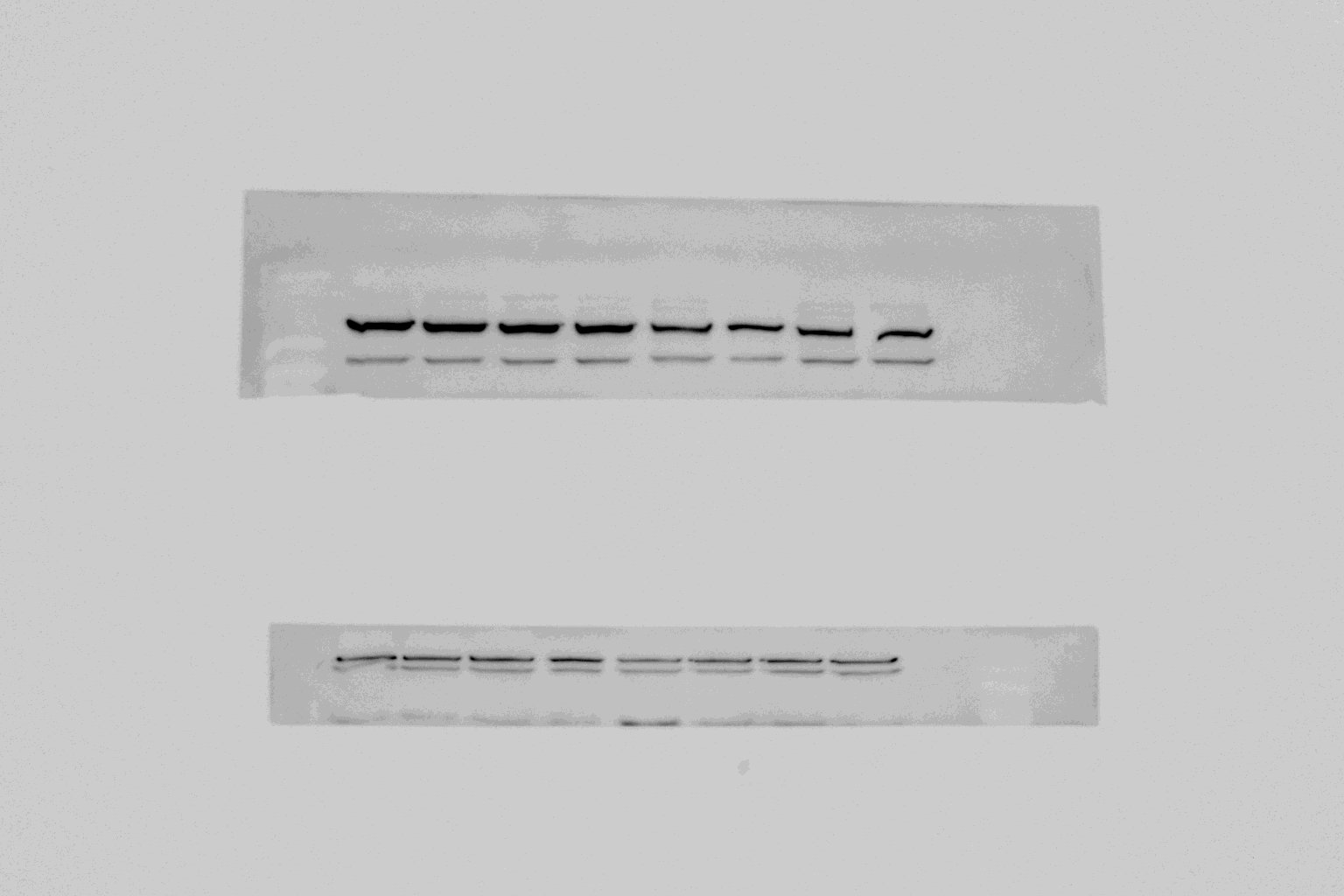

Supplement: Supplementary file 1 [file biomolecules-13-01277-s001.zip › Supplementary File S1/p35 y p42 Atg7/20180111_1224_3.jpg]

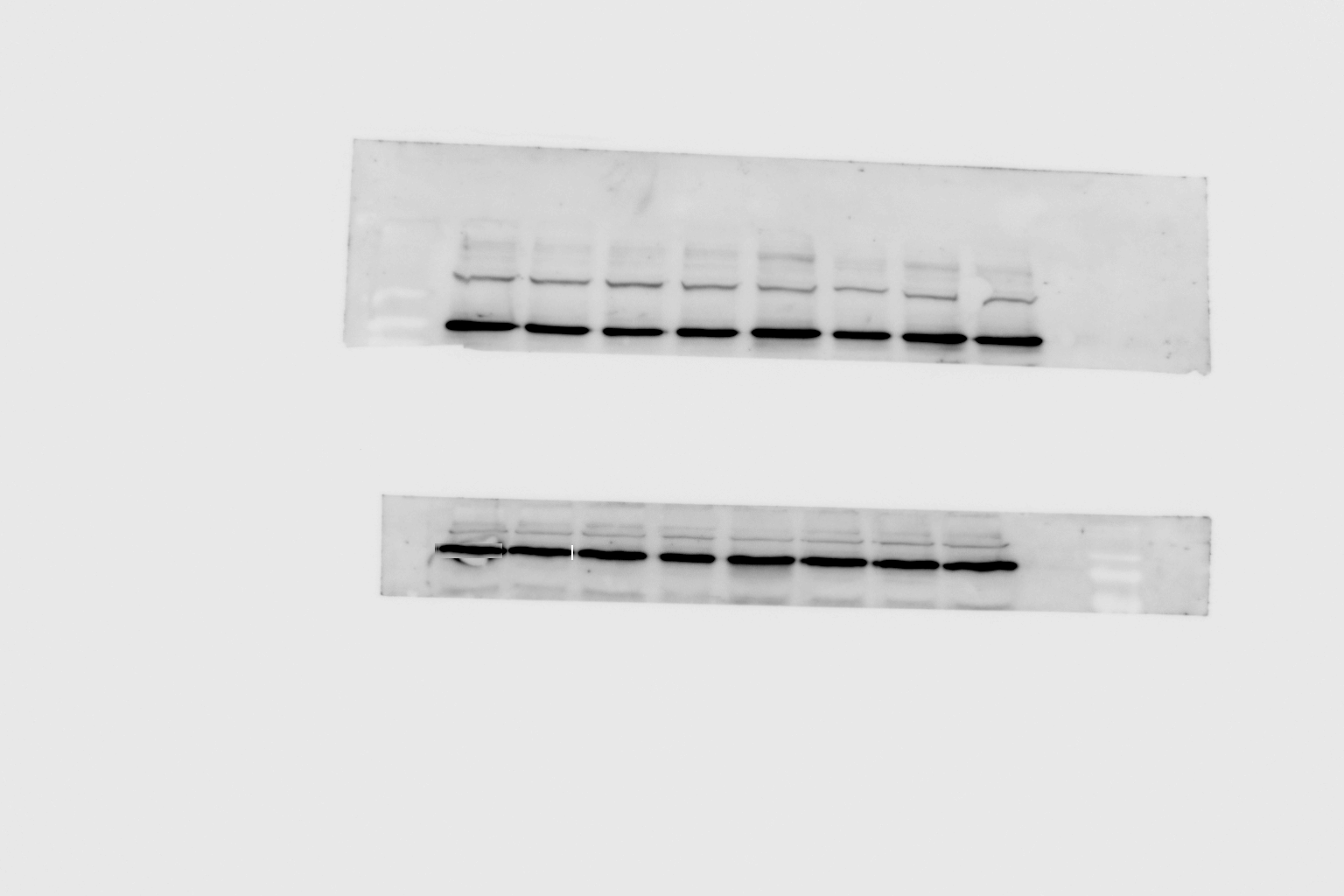

Supplement: Supplementary file 1 [file biomolecules-13-01277-s001.zip › Supplementary File S1/p35 y p42 BECN/20180112_1159_1.tif]

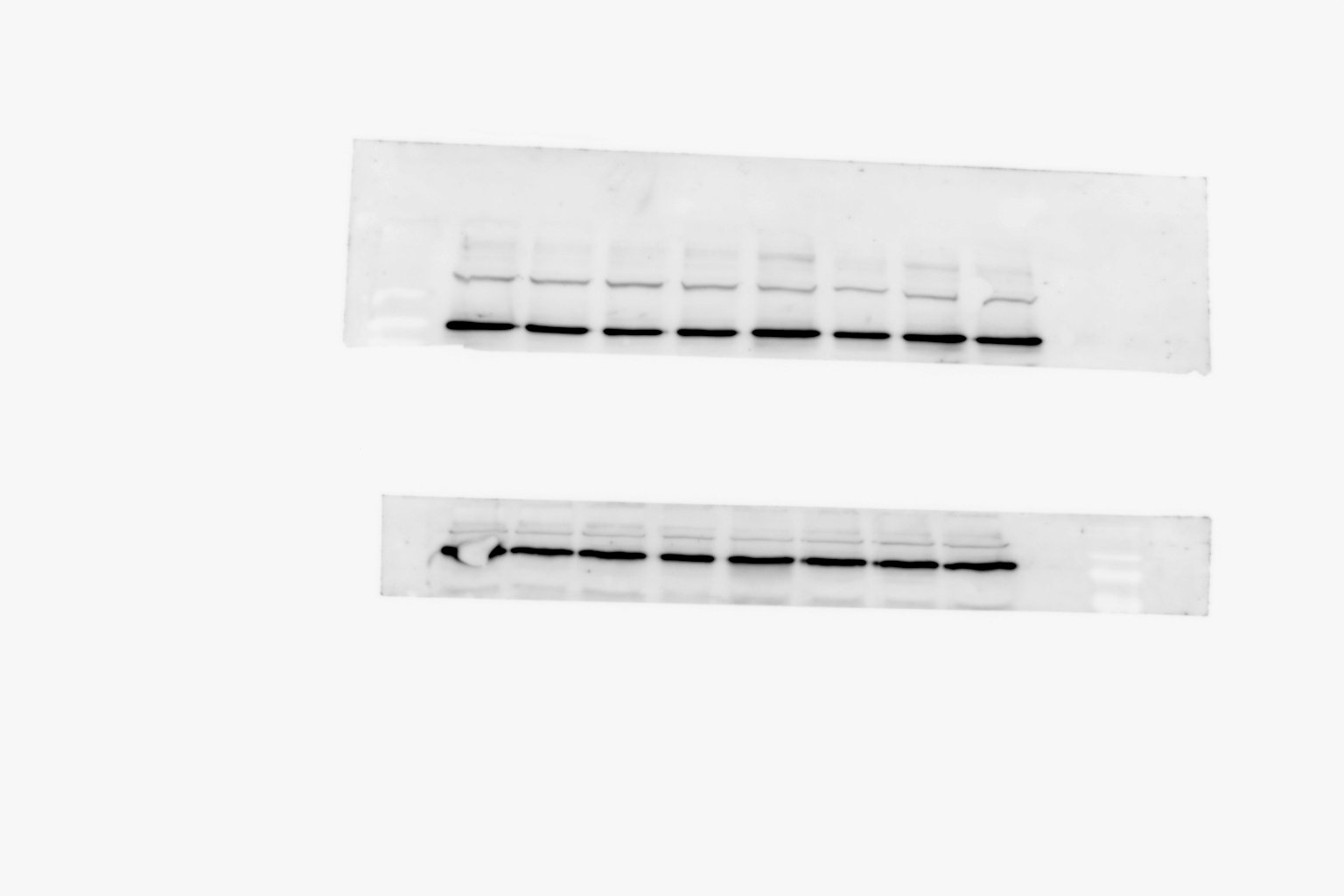

Supplement: Supplementary file 1 [file biomolecules-13-01277-s001.zip › Supplementary File S1/p35 y p42 BECN/20180112_1159_2.jpg]

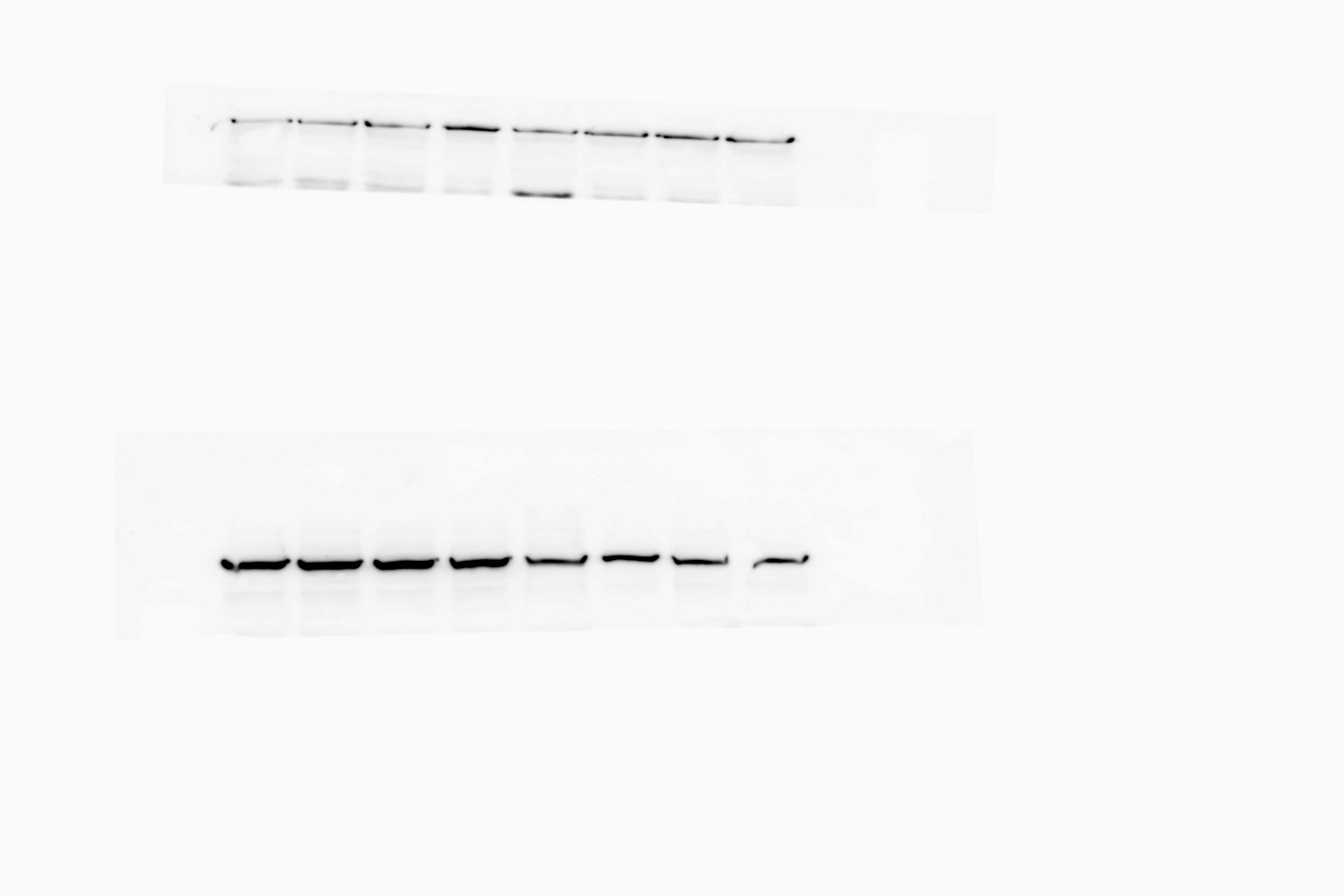

Supplement: Supplementary file 1 [file biomolecules-13-01277-s001.zip › Supplementary File S1/p35 y p42 L2A/20180110_1320_3.jpg]

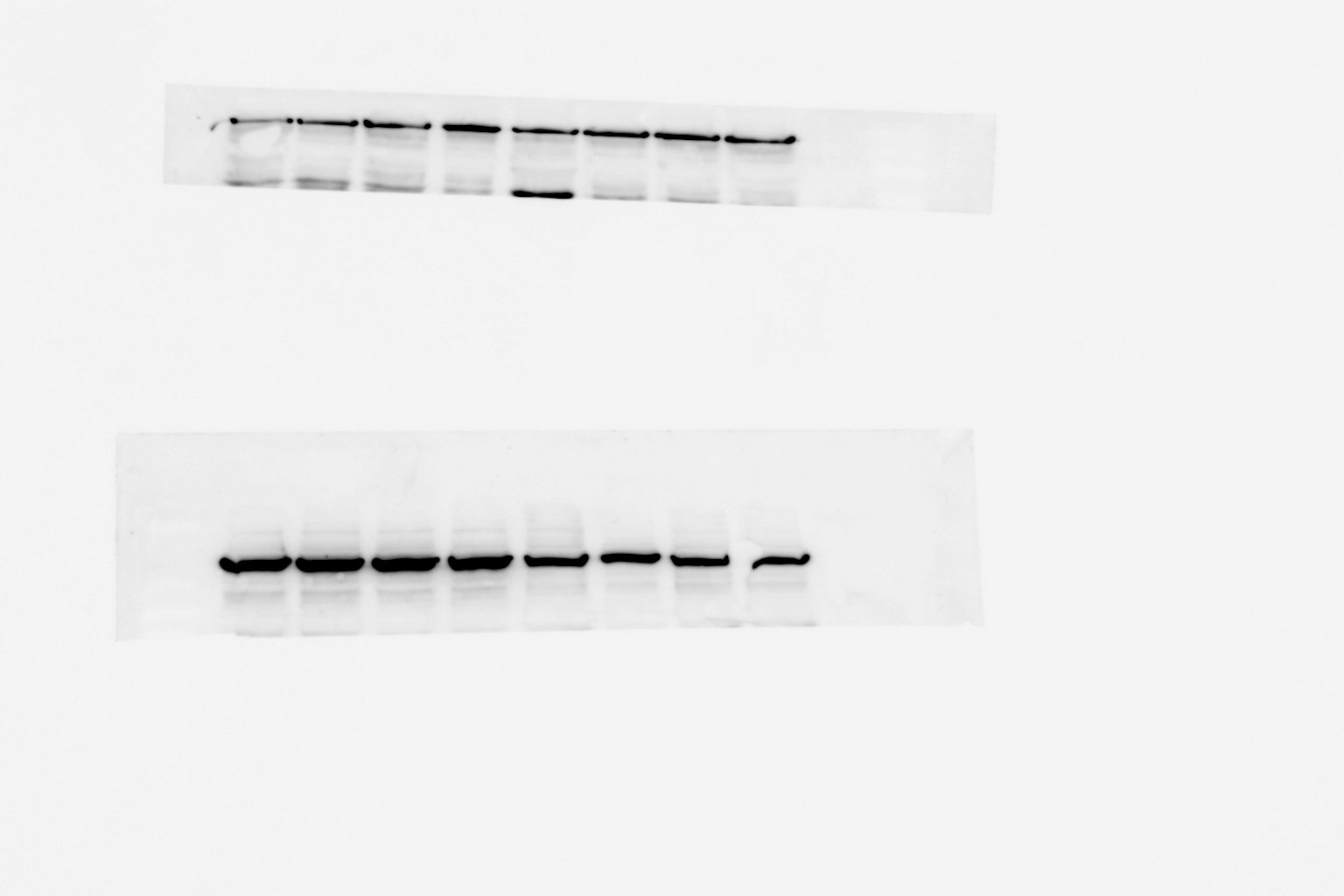

Supplement: Supplementary file 1 [file biomolecules-13-01277-s001.zip › Supplementary File S1/p35 y p42 L2A/20180110_1320_4.jpg]

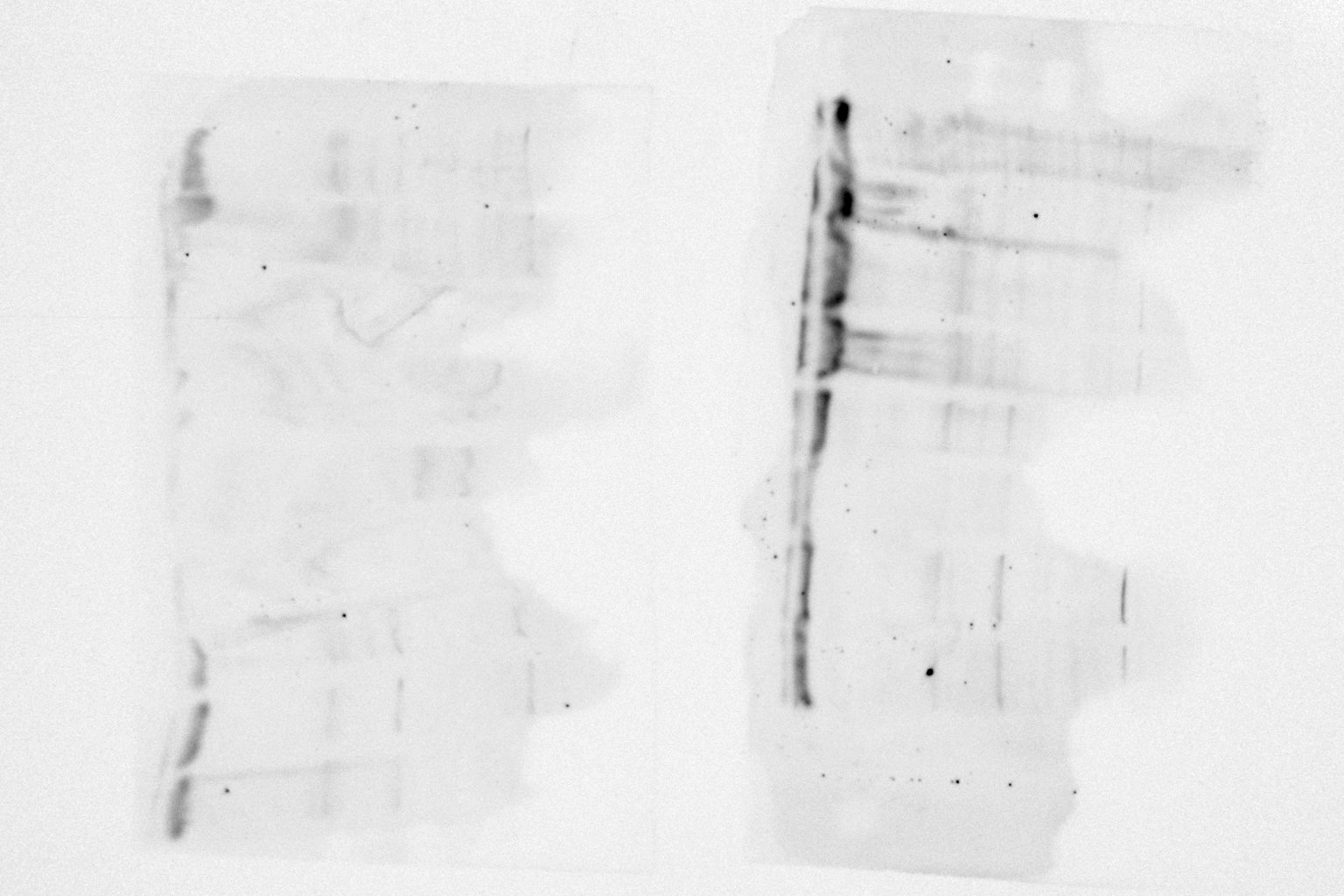

Supplement: Supplementary file 1 [file biomolecules-13-01277-s001.zip › Supplementary File S1/p35 y p42 LC3 Bueno/20180125_1257_8.jpg]
